# Understanding the Profile of Tuberculosis and Human Immunodeficiency Virus Coinfection: Insights from Expanded HIV Surveillance at a Tuberculosis Facility in Durban, South Africa

**DOI:** 10.1155/2014/260329

**Published:** 2014-04-07

**Authors:** Rubeshan Perumal, Nesri Padayatchi, Kogieleum Naidoo, Stephen Knight

**Affiliations:** ^1^Centre for the AIDS Programme of Research in South Africa (CAPRISA), University of KwaZulu-Natal, Durban 4000, South Africa; ^2^Department of Public Health Medicine, University of KwaZulu-Natal, Durban 4000, South Africa

## Abstract

*Background*. Expanded HIV surveillance in TB patients forms part of the World Health Organization framework for strategic collaborative activity. Surveillance helps understand the epidemiology of the local dual epidemic and enables design of a tailored response to these challenges. *Methods*. We conducted an observational, cross-sectional study of anonymous unlinked HIV testing for 741 consecutive TB suspects attending an urban TB facility during a seven-week period in 2008. *Results*. A total of 512 patients were found to have TB. The mean age was 35.7 years, and 63% were male. The prevalence of HIV was 72.2% (95% CI: 68.2–75.9) in all TB cases, 69.8% (95% CI: 65.3–74.2) in pulmonary tuberculosis (PTB), 81.6% (95% CI: 72.9–90.3) in extrapulmonary disease, and 66.8% (95% CI: 60.7–72.9) in those without TB disease. HIV prevalence in TB patients was higher in females than males and in younger age groups (18–29 years). The sex ratio of PTB patients correlated with the sex ratio of the prevalence of HIV in the respective age groups (*P* < 0.05). *Conclusion*. The use of a rapid HIV test performed on sputum anonymously provides an opportunity for HIV surveillance in this high-burdened setting, which has the potential to lend valuable insight into the coepidemics.

## 1. Background

In South Africa, the global epicenter of both tuberculosis (TB) and human immunodeficiency virus (HIV), the menacing convergence of these diseases: one viral and one bacterial, one emergent, and one ancient, presents a near insurmountable challenge to health, social, economic, and developmental welfare [1]. These dual epidemics threaten to undo the health care gains of the past decades in an already ailing health care system. Women now account for nearly a half of all global infections and 77% of all women living with HIV are in sub-Saharan African [2]. Acquired immune deficiency syndrome (AIDS) accounts for more deaths in women than all causes of maternal mortality combined [2]. While the global incidence of HIV is decreasing, new infections among young women aged 15 to 24 years have steadily increased in South Africa, which is due to multifactorial and complex biological, social, economic and behavioural factors.

The World Health Organization (WHO), in an effort to address the challenges of HIV associated TB, has proposed a framework of strategic collaborative activities for TB and HIV services which foster a more comprehensive approach to the synergistic dual epidemics [3]. In settings of generalized HIV and AIDS epidemics, like South Africa, WHO recommends expanding HIV surveillance to include TB patients.

Anonymous unlinked testing for HIV by means of a noninvasive sputum antibody test has been proposed as a means of HIV surveillance within South African TB facilities. This approach is cost-effective, sustainable, and feasible for rapid expansion of HIV surveillance in South Africa.

Comprehensive HIV surveillance is central to understanding the epidemic, the unique challenges in each country, the trajectory of the epidemic, resultant morbidity and mortality, and the impact of interventions. The WHO initiated unlinked, anonymous testing (UAT) through sentinel seroprevalence surveys amongst antenatal attendees as the global standard for HIV surveillance where heterosexual transmission is dominant. In UAT, blood drawn for routine purposes in the health care setting are stripped of patients-identifiers, unlinked from patient records, and tested for HIV [4]. This method has the primary advantage of eliminating nonresponse bias typically associated with other HIV surveys where consent is required. Since then this method has been the universally adopted standard throughout the world and serves as the methodological basis for comparing the prevalence of HIV across the globe, especially in generalized epidemics.

As an additional surveillance activity to antenatal surveys, HIV surveillance among TB patients is strongly recommended, especially in countries like South Africa where TB and HIV are deleteriously intertwined coepidemics. Expanding HIV surveillance to include TB patients will serve as an indicator of the level and maturity of the epidemic, as well as an indicator of the effect of HIV on health services [5].

The WHO guidelines for second generation HIV surveillance reiterate the need for additional data on HIV trends, not obtained from antenatal clinic surveys [6]. These guidelines promote a comprehensive HIV surveillance system, to better understand HIV trends and the factors that drive the epidemic in a country, by focusing on flexible surveillance in specific high risk populations to improve prevention and care activities [6].

The specific objectives of HIV surveillance among TB patients in generalized HIV epidemics are (a) to inform the targeting of resources and planning of activities for people with HIV associated TB and for monitoring the effectiveness of these activities; (b) to increase political, professional, and civil society awareness of coepidemics; (c) to assess the need for collaboration between HIV and TB programmes on formulation and implementation of a joint TB-HIV strategy; (d) to provide information on the HIV and AIDS epidemic and its impact on TB patients; and (e) to quantify the need for providing antiretroviral (ART) to TB patients [7].

One of the main challenges in conducting HIV surveillance among TB patients is the ethical concerns primarily surrounding the use of anonymous, unlinked testing. In settings with increasing availability of ART, there is considerable controversy about the ethical validity of anonymously testing for HIV where emphasis should be placed on counseling and testing for HIV and the provision of ART to patients who require it [8]. However, in general, testing without informed consent, for the purpose of surveillance, has been considered ethical if anonymous, unlinked testing is employed [7]. Moreover, in generalized epidemics with a high burden of TB-HIV coinfection, such as South Africa, the benefits of surveillance justify its inclusion as an additional method of surveillance. HIV surveillance among TB patients in the South African context using unlinked, anonymous testing will increase the epidemiological utility of the data by eliminating participation bias and will occur as part of a response to the public health emergency characterised by TB-HIV coinfection.

An existing challenge to anonymous, unlinked testing for HIV among TB patients is the lack of routine blood testing in TB on which HIV testing can piggyback. This has fuelled concerns as to whether the inclusion of routine bloods in TB will be driven by the covert intention to introduce anonymous, unlinked HIV testing, rather than out of a real need in the patient's interest. The use of novel methods for HIV testing on sputum specimens is rapidly gaining momentum in response to such concerns. Sputum, in contrast to blood, is routinely collected from TB patients, discarded after its use, and is being shown by an expanding evidence base to be useful in the diagnosis of HIV [7, 9–12]. The main ethical proviso regarding HIV surveillance among TB patients is that consent may only be omitted if samples are taken from routine specimens and testing is anonymous and is not linked to patient identifiers. Additionally, all TB patients included in HIV surveillance activities should have access to Voluntary Counseling and Testing (VCT) for HIV infection.

This paper explores anonymous unlinked HIV testing of TB suspects as a means to understand the local coinfection epidemic and the need to integrate services.

## 2. Method

### 2.1. Study Design

An observational, analytic, cross-sectional study design was used.

### 2.2. Study Site

The survey was conducted at an out-patient tuberculosis facility in Durban, South Africa. The clinic, situated in the central business district adjacent to a transportation nodal point, provides diagnosis and treatment of TB for those who reside or work in Durban. Sputum microscopy and HIV testing are conducted in the on-site laboratory.

### 2.3. Study Sample

The study sample included all adult patients presenting for TB diagnostic services who provided a sputum specimen during the study period.

### 2.4. Laboratory Method

OraQuick HIV 1/2 antibody testing was performed by a trained technician as per manufacturer guidelines on all sputum specimens prior to discard, irrespective of its suitability for TB microscopy [13].

### 2.5. Statistical Analysis

All data was processed and analysed using SAS Software (SAS 9.1.3, SAS Institute, Inc., Cary NC). For all statistical comparisons, the 5% significance level was used; correspondingly, 95% confidence intervals were used to describe effect size. All data was assessed for normality, and nonparametric tests were used where necessary. HIV prevalence for TB cases and suspects was calculated and selected demographic risk factors associated with the patients HIV status were measured using chi-squared tests for categorical data (participants age categories, gender, and visit type) and analysis of variance techniques for continuous variables (age).

### 2.6. Permissions

Ethical approval was obtained from the Biomedical Research Ethics Committee of the Africa University of KwaZulu-Natal (Reference number BFC031/08). Permission to conduct the study at a municipal health facility was obtained from the eThekwini Municipality Health Unit.

## 3. Results 

### 3.1. Age and Sex Characteristics of the Study Sample

The mean age of the survey population was 35.7 years (SD ± 11.3 years). The mean ages of males and females were similar (36.9 versus 33.6 years, *P* = 0.234). Two-thirds (467/741) of the study sample were male. Of the total, 622 (84%) were between 20 and 50 years old, an identical proportion in both males (84%) and females (84%) (Table 1). Only 15 (3%) of the males were younger than 20 years old, compared to 21(8%) of the females (*P* < 0.05). There was a significantly higher proportion of males than females over 50 years of age (13% versus 8%, *P* < 0.05).

### 3.2. HIV Prevalence

**Figure 1:**
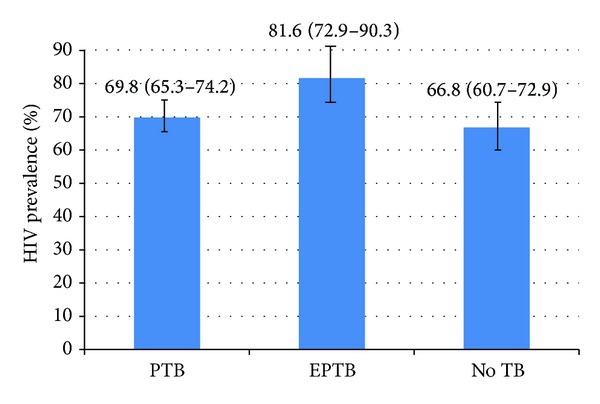
HIV prevalence (95% confidence intervals) in those with pulmonary tuberculosis (*n* = 407), extrapulmonary tuberculosis (*n* = 105), and without tuberculosis disease (*n* = 229) at the clinic, 2008.

A total of 229 (31%) TB suspects did not have a diagnosis of TB confirmed, of which 153(66.8%, 95% CI: 60.7–72.9) tested sputum HIV antibody positive. The balance of the study population of TB suspects, 512 (69%), was diagnosed with TB, of which 369 (72.1%, 95% CI: 68.2–76.0) were HIV positive. There was no statistical difference in HIV prevalence between these two groups (*P* = 0.15). The prevalence of HIV was 69.8% (95% CI: 65.3–74.2) and 81.6% (95% CI: 72.9–90.3) in those with pulmonary and extrapulmonary TB, respectively (Figure 1).

**Figure 2:**
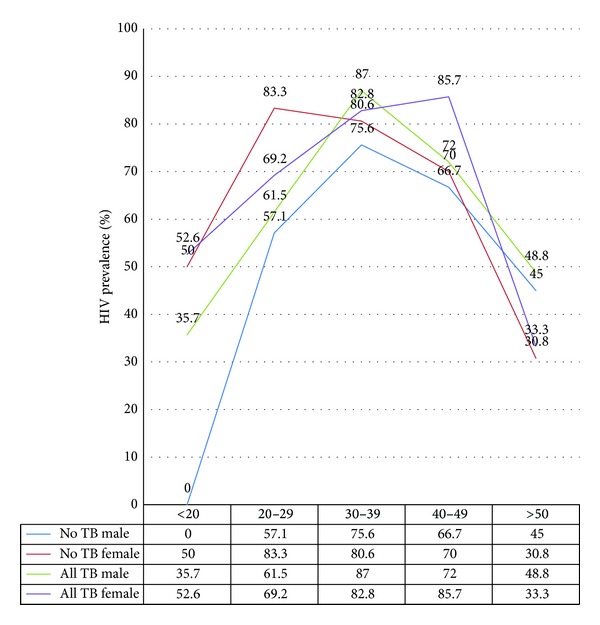
HIV prevalence by age group, sex, and TB status in the study cohort.

The HIV prevalence in TB suspects accessing the clinic varies by age, sex, and by the presence or absence of TB disease. In those without TB disease, the age- and sex-specific HIV prevalence reflects a similar pattern to the general population. Females have a higher age- specific HIV prevalence in all age groups except those 50 years and older, have an earlier peak prevalence than males (20–29 years versus 30–39 years), and have a higher peak prevalence (83.3% versus 75.6%) than males (Figure 2).

In those diagnosed with TB, the age-specific HIV prevalence in females was higher than in males in age groups under 20, 20–29, and 40–49. However, the peak prevalence (86%) of HIV in females occurred in the 40–49 years age group as compared to a peak male HIV prevalence of 87% in the 30–39 years age group. Females with and without TB (overall) have higher age- specific prevalence than males with and without TB in the younger age groups, while the males have higher age-specific prevalence than females in those 50 years and older.

The male-to-female sex ratio in all patients with pulmonary TB ranges from 0.79 in the under 20 years age group, to 5.3 in the greater than or equal to 50 years age group (Table 2). The representation of males increases across the increasing age groups. In HIV associated pulmonary TB, the male-to-female ratio ranges from 0.38 in the under 20 years age group to 7.5 in those 50 years and older. The proportion of females increases in the age groups less than 20 years, 20 to 29 years, and 40 to 49 years. In these age groups, the prevalence of HIV among females is higher than the HIV prevalence among males. There exists a significant positive correlation (*r* = 0.87, *P* < 0.05) between the change in the sex ratio between all those with PTB and PTB associated HIV and the sex ratio of HIV prevalence across the age groups.

## 4. Discussion

The primary objective of this study was to provide an estimate for the prevalence of HIV among attendees at an urban TB facility in Durban, South Africa, using a novel sputum-based HIV antibody test. The paucity of such information in the past has limited the ability of health services to exploit the opportunities presented by the changing epidemiology of TB and HIV in this setting. Analysis of the age and sex distribution of HIV associated TB in this study reflects the impact of a maturing HIV epidemic on TB in South Africa. Only 37% of TB patients were female, and the male-to-female ratio of 1.7 is lower than global and historic trends [14, 15]. Globally, in 2007, there was a 2 : 1 male-to-female ratio of cases notified to public health authorities [16]. Nonetheless, in low income countries, TB kills more women than all causes of maternal mortality combined. There was more pulmonary TB in females than in males under 30 years in this study. This increased burden of TB in young women has been attributed to biological, social, and methodological factors. Young women have up to 34% higher risk of progression to TB disease than men up to the age of 30 years initially attributed to reduced immunity associated with the stresses of pregnancy [16]. However, this hypothesis was not supported by a case-control study which demonstrated that pregnancy was not an exposure associated with TB disease [17]. In addition, young women have lower cumulative TB infections in adolescence, inferring that new postadolescence infection might be significantly greater amongst women. New infection with TB carries a greater likelihood to progress to TB disease, and to do so more rapidly. Females are more likely to encounter health services during their reproductive years, increasing their likelihood of having TB diagnosed. Similarly, the lower prevalence of TB disease in older women compared to men may reflect their differential access to healthcare due to a combination of complex social, economic, cultural, and logistical factors. This may be particularly true in settings such as South Africa where active case finding is too poorly implemented to rectify possible undernotification of women and their possible underrepresentation at health facilities. This age-sex trend of TB is similar to WHO estimates for other African countries with a high HIV prevalence where there are more female than male cases notified up to 25 years. The estimate was confirmed in this high HIV-burdened setting, with a male-to-female ratio of 0.9 in cases of pulmonary TB under 30 years of age.

The disproportionate burden of HIV on young women has largely been attributed to intergenerational sex of young women with older men, who are likely to have multiple concurrent sexual partners [2, 18, 19]. Young women have a 7-fold increased risk of acquiring HIV when in relationships with men between 5 and 7 years older [20–23]. The exact reasons for the increased risk of acquiring HIV in young women are not yet understood but include increased transmissibility from males to females, anatomy, condom use, sexual networking, poverty, education, economic autonomy, power imbalances between men and women, gender based violence, access to sexual and reproductive health services, and political and sociocultural issues [2]. The pattern of a higher HIV prevalence among young women in comparison to men in the same age group is now being reflected in the age-specific trends of TB. The impact of HIV on TB is reflected in the age-specific HIV prevalence figures generated by this study and confirms that HIV is shaping the evolving epidemiology of TB in this setting.

With nearly 70% of all TB patients being coinfected with HIV, it is not surprising that the age and sex distribution of TB is increasingly resembling the distribution of HIV in this region of hyperendemicity. The male-to-female ratio among HIV negative individuals was 1.98 while the male-to-female ratio among those living with HIV drops to 1.58. The increased representation of females in comparison to males in HIV associated pulmonary TB is significantly correlated with the sex-specific HIV prevalence figures in those age groups. There is a clear indication that HIV is driving the increased representation of females among TB patients with age groups in which females have the higher HIV prevalence containing a greater number of females with TB. A previous study in this setting demonstrated a significantly higher burden of TB disease among women in the 20 to 29 years age group, in keeping with predictions by mathematical models on the impact of HIV on TB in areas of dual hyperendemicity [24]. Whether this is due to biological or structural reasons is uncertain, but the greater representation of females at TB facilities in high HIV settings has multiple programmatic implications. A growing recognition of the gradually changing face of TB in this setting will allow for the exploitation of the opportunities presented by this evolving epidemiology. The unique opportunity to seek out synergies between TB-HIV and women's health services must be seized. The broadening of services may serve to appropriately orientate the existing TB services with regards to the changing demographic profile of patients to augment women's health services, to respond to the need for comprehensive primary health care services, and, most importantly, to acknowledge the presence of multiple pathologies in an individual patient that cannot adequately be addressed by a vertical programme design.

## 5. Conclusions

The demographic profile of TB patients mirrors the patterns observed in those living with HIV and suggests that HIV infection does fuel the TB epidemic in this setting. The increased number of young women, in particular, at TB services presents the opportunity to design more demographically sensitive TB services which include a more comprehensive health service package. The benefit of including women friendly reproductive health services at TB facilities requires further evaluation. Overall, this survey demonstrates the possibility of using a novel HIV antibody assay on sputum and an anonymous survey design for achieving additional HIV surveillance in a generalized HIV epidemic with TB endemicity. A better understanding of both of these epidemics and how they interact is crucial to optimise a consolidated comprehensive public health response. The richness of data that is generated is a clear indication of the potential benefits that may accrue from better understanding the confluence of these dual epidemics. The idea of an expanded surveillance programme for HIV in TB facilities is a direct response to a call from WHO to optimize the position of health systems to understand, appreciate, and react to the emerging destructive synergy between TB and HIV epidemics.

This study has highlighted that the problem of TB-HIV coinfection is not a homogenous entity but rather a composite of many complex subepidemics. This is largely due to the various drivers of HIV and TB which have differential effects on various strata of the population, most notably subpopulations by age and sex. A striking feature of the TB-HIV coepidemic is the direct influence that the epidemiology of HIV has had on the evolving TB epidemic, with young women representing a significant proportion of TB facility attendees. The majority of TB patients are now also in the economically active age group. The hyperendemicity of TB and HIV in South Africa coupled with the existing demographic distribution of the coepidemics has serious implications for the social, health, and economic status of the nation and threatens to be a significant limitation to national growth and development. Vertical programme designs have failed to demonstrate that they are capable of responding to the synergism of multiple morbidities in individuals. A more integrated approach is more attractive as it promises the possibility of increasing clinical and operational efficiency. This is the central theme of the WHO call for closer collaboration between TB and HIV services. However, the guiding tenet of all health care initiatives is to ensure that programmes are responsive to the needs of patients and that any intervention is tailored to respond to those needs while being informed by the unique epidemiology of the local epidemic. The local epidemic, as demonstrated in this study, is characterised by a broad cross-section of patients, with the majority of coinfected individuals in the economically active age group, and with the majority of women in their reproductive years. There are therefore several opportunities to maximize the impact of any healthcare contact with these individuals when they present to a TB facility. Sexual health services, in particular, would be well positioned in TB facilities and could aim to provide sexual health education, positive prevention, condom promotion, screening for sexually transmitted diseases, and the treatment thereof.

This study has confirmed that the burden young women face in terms of their heightened vulnerability for HIV infection has been translated to an increased vulnerability for TB. Existing TB programmes do not adequately reflect the needs of young women in their design. Women's health and basic reproductive health services would have a significant target audience at TB facilities. This might include access to family planning, Pap smear evaluations, and targeted interventions for young women such as microbicide gels in future. A more comprehensive service may lead to greater public access, more equitable access for all patients, a more convenient and satisfying service, and better health overall. New TB service design must begin to reflect the presence of young women as a significant group burdened by the disease.

## Figures and Tables

**Table 1:** Age and sex characteristics of study survey cohort.

Age group	Female	Male	Total	*P* value
Years	*n* (%)	*n* (%)	*n* (%)	
<20	21 (7.7)	15 (3.2)	36 (4.9)	<0.05
20–29	89 (32.5)	100 (21.4)	189 (25.5)	<0.05
30–39	94 (34)	183 (39.2)	277 (37.4)	0.21
40–49	48 (17.5)	108 (23.1)	156 (21.0)	0.09
≥50	22 (8.0)	61 (13.1)	83 (11.2)	<0.05
Total	**274 (37.0) **	**467 (63.0)**	**741 (100)**	

Overall age (*n* = 741) mean: 35.7 years (95% CI: 34.9–36.5).

Males age (*n* = 467) mean: 36.9 years (95% CI: 35.9–37.9).

Females age (*n* = 274) mean: 33.6 years (95% CI: 32.2–35).

**Table 2:** Correlation between the change in the male-to-female sex ratio in all PTB and HIV associated PTB and ratio of sex-specific HIV prevalence across age groups.

Age group	Total M : F	HIV + M : F	ΔM : F	Male : female
	(all PTB)	(HIV + PTB)	(change in ratio)	HIV prevalence
<20	0.8	0.4	−0.4	0.5
20–29	1.0	0.9	−0.1	0.9
30–39	2.5	2.6	0.1	1.0
40–49	2.7	2.4	−0.3	0.9
≥50	5.3	7.5	2.2	1.4

Pearson's correlation, one-tailed, *r* = 0.87, df = 3, and *P* < 0.05.
